# Characterization of the Transcriptome and Gene Expression of Brain Tissue in Sevenband Grouper (*Hyporthodus septemfasciatus*) in Response to NNV Infection

**DOI:** 10.3390/genes8010031

**Published:** 2017-01-13

**Authors:** Jong-Oh Kim, Jae-Ok Kim, Wi-Sik Kim, Myung-Joo Oh

**Affiliations:** Department of Aqualife Medicine, College of Fisheries and Ocean Science, Chonnam National University, Yeosu 550-749, Korea; jongoh.kim77@gmail.com (J.-O.K.); hoy0924@naver.com (J.-O.K.); wisky@jnu.ac.kr (W.-S.K.)

**Keywords:** nervous necrosis virus (NNV), sevenband grouper, transcriptome, next generation sequencing (NGS), differential expressed genes (DEGs)

## Abstract

Grouper is one of the favorite sea food resources in Southeast Asia. However, the outbreaks of the viral nervous necrosis (VNN) disease due to nervous necrosis virus (NNV) infection have caused mass mortality of grouper larvae. Many aqua-farms have suffered substantial financial loss due to the occurrence of VNN. To better understand the infection mechanism of NNV, we performed the transcriptome analysis of sevenband grouper brain tissue, the main target of NNV infection. After artificial NNV challenge, transcriptome of brain tissues of sevenband grouper was subjected to next generation sequencing (NGS) using an Illumina Hi-seq 2500 system. Both mRNAs from pooled samples of mock and NNV-infected sevenband grouper brains were sequenced. Clean reads of mock and NNV-infected samples were de novo assembled and obtained 104,348 unigenes. In addition, 628 differentially expressed genes (DEGs) in response to NNV infection were identified. This result could provide critical information not only for the identification of genes involved in NNV infection, but for the understanding of the response of sevenband groupers to NNV infection.

## 1. Introduction

Grouper is one of the highest valued marine fish and has become an important species in the aquaculture industry of various Asian countries. In Korea, sevenband grouper (*Hyporthodus septemfasciatus*) is one the favorite grouper fish consumed. Its production rate is increasing. However, viral nervous necrosis (VNN) causes high mortality, especially at the larval and juvenile stage of sevenband groupers during the summer season, which has caused vast economic losses [1].

Viral Nervous Necrosis is a serious disease in the world aquaculture industry [2,3,4]. Firstly, it was reported in bigeye trevally (*Caranx sexfasciatus*) in the 1980s and since then it has been reported in over twenty species [2,3,4]. The infected fish are usually swimming abnormally and having vacuolization and necrosis of the central nervous system in the brain [3]. In Korea, mass mortalities caused by VNN have been reported from various cultured marine fish such as sevenband grouper (*Hyporthodus septemfasciatus*), rock bream (*Oplegnathus fasciatus*), red drum (*Sciaenops ocellatus*) and olive flounder (*Paralichthys olivaceus*) since 1990 [5,6,7].

Nervous necrosis virus (NNV), the causative agent of VNN, has non-enveloped icosahedral structure and belongs to the family *Nodaviridae* (genus *Betanodavirus*). Its genome contains two single-stranded positive senses RNA: RNA1 (approximately 3.1 kb in length) encodes an RNA-dependent RNA polymerase for viral replication, whereas RNA2 (1.4 kb) encrypts a protein α. During RNA replication, a sub-genomic RNA3 was produced from the 3′ terminus of RNA1 that encodes non-structural proteins B2 [8]. Betanodaviruses have five genogroups based on the T4 region sequence of RNA2 as barfin flounder nervous necrosis virus (BFNNV), red-spotted grouper nervous necrosis virus (RGNNV), striped jack nervous necrosis virus (SJNNV), tiger puffer nervous necrosis virus (TPNNV) [9], and turbot nervous necrosis virus [10].

Although virological and genetic characterizations of NNV have been reported, its infection mechanisms and disease outbreak mechanisms remain unclear. Therefore, systematic approaches are needed to determine its infection mechanisms.

Recently, rapid progress has been made in next generation sequencing (NGS) technology and bioinformatics. They are powerful tools for transcriptome analysis. RNA-Seq by NGS is one of the most useful methods to survey the landscape of a transcriptome since it produces millions of data on gene expression. Numerous novel genes and unraveling expression profiles related phenotypic changes, such as development stages, were identified using RNA-Seq [11,12]. Several studies using RNA-Seq have reported the immune relevant genes of fish after pathogen challenges. For example, zebrafish (*Danio rerio*) [13], orange-spotted grouper (*Epinephelus coioides*) [14], large yellow croaker (*Pseudosciaena crocea*) [15], turbot (*Scophthalmus maximus*) [16], Japanese sea bass (*Lateolabrax japonicus*) [17] and common carp (*Cyprinus carpio*) [18] in response to pathogen challenges have been analyzed.

In this study, we analyzed the brain transcriptome of sevenband grouper in response to NNV infection. We annotated the transcripts using Gene Ontology (GO) and non-redundant (nr) databases of GenBank. In addition, we obtained differential expression genes (DEGs) between sevenband grouper brain infected by NNV and non-infected sevenband grouper brain. To the best of our knowledge, this is the first study that reports the transcriptome of brain tissue of NNV-infected sevenband grouper by RNA-Seq. Our transcriptome analysis data will provide valuable genomic information to determine the functional roles of these genes related to NNV infection and VNN outbreak in the future.

## 2. Materials and Methods

### 2.1. Ethics Statment

The experiments using sevenband grouper were carried out in strict accordance with the recommendations of the Institutional Animal Care and Use Committee of Chonnam National University (Permit Number: CNU IACUC-YS-2015-4).

### 2.2. Experimental Fish and Virus 

Sevenband groupers were purchased from an aqua-farm without history of VNN occurrence. Prior to experiment, brain tissues of 10 fish were randomly sampled from the aqua-farm and subjected to reverse transcription polymerase chain reaction (RT-PCR) analysis to determine betanodavirus infection according to a previous report [9]. The NNV (SGYeosu08) [19] isolated from sevenband grouper aqua-farm in Korea was propagated in the striped snakehead (SSN-1) cell line. SSN-1 cells were grown at 25 °C in Leibovitz L-15 medium (Sigma Aldrich, St. Louis, MO, USA) containing 10% fetal bovine serum (FBS, Gibco, Waltham, MA, USA), 100 µg/mL streptomycin and 150 U/mL penicillin G. NNV was inoculated onto SSN-1 cell monolayer and incubated at 25 °C for the virus propagation. After the cells were completely lysed, virus titer was calculated by the Reed and Muench method [20]. Viral samples were aliquoted into small volumes and stored at −80 °C until use.

### 2.3. Virus Challenge

Twenty fish (mean body weight, 20 g) were reared in two 40 L tanks (*n* = 10/tank) at 25 °C. Ten fish were intramuscularly injected with NNV at doses of 10^3.8^ TCID50/fish. The remaining 10 fish were injected with L15 medium as a control. The challenged fish were observed daily. The NNV infected fish died from day 3 after infection and showed 100% of cumulative mortality after 1 week. The moribund fish at days 3 and 4 were selected for sampling. Brain tissues of three of ten challenged fish from mock and the virus-challenge group were collected and pooled for NGS analysis, respectively. 

### 2.4. Next Generation Sequencing of Transcriptome

To obtain high-throughput transcriptome data of sevenband grouper, complementary DNA (cDNA) libraries were prepared for 100 bp paired-end sequencing using a TruSeq RNA Sample Preparation Kit (Illumina, San Diego, CA, USA) according to the manufacturer’s protocols. They were then paired-end (2 × 100 bp) sequenced using an Illumina HiSeq2500 system (Illumina, San Diego, CA, USA).

### 2.5. Transcriptome Assembly and Functional Annotation

Prior to de novo assembly, paired-end sequences were filtered and cleaned using an NGS QC toolkit [21] to remove low quality reads (Q < 20) and adapter sequences. In addition, bases of both ends less than Q20 of filtered reads were removed additionally. This process is to enhance the quality of reads due to mRNA degradation in both ends of it as time goes on [22]. Only high quality reads were used for de novo assembly performed by Trinity (version 20130225) using default values [23]. To remove the redundant sequences, CD-HIT-EST [24] was used. NCBI Blast (version 2.2.28) was applied for the homology search to predict the function of unigenes. The function of unigenes was predicted by Blastx to search all possible proteins against the NCBI Non-redundant (NR) database (accessed on 17 July 2013). The criterion regarding significance of the similarity was set at Expect-value less than 1 × 10^−5^.

### 2.6. Differentially Expressed Genes Analysis

After obtaining the assembled transcriptome data using Trinity, gene expression level was measured with RNA-Seq by Expectation Maximization (RSEM), a tool for measuring the expression level of transcripts without any information on its reference [25]. The TCC package was used for DEG analysis through the iterative DEGES/DEseq method [26]. Normalization was progressed three times to search meaningful DEGs between comparable samples [27]. The DEGs were identified based on the *p*-value of less than 0.05.

### 2.7. GO Enrichment of Differentially Expressed Genes

The GO database classifies genes according to the three categories of Biological Process (BP), Cellular Component (CC) and Molecular Function (MF) and provides information on the function of genes. To characterize the identified genes from DEG analysis, a GO based trend test was carried out through the Fisher’s exact test. Selected genes with *p*-values of less than 1 × 10^−5^ were regarded as statistically significant.

### 2.8. Data Deposition

All the raw read files were submitted to the sequence reads archive (SRA), NCBI database (accession number—SRR5091816).

## 3. Results

### 3.1. Sequence Analysis of the Transcriptome

**Figure 1:**
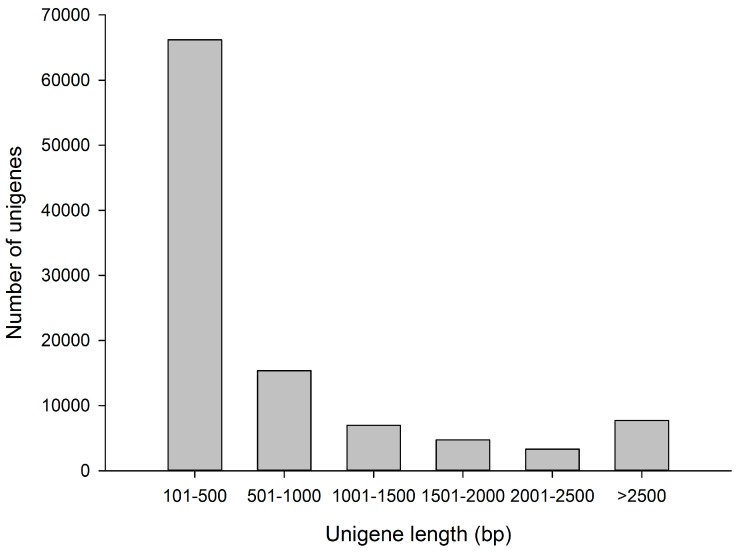
Length distribution of unigenes obtained from transcriptome analysis.

Sequencing of the two libraries (mock and NNV-infected brain tissue) using the Illumina Hiseq 2500 platform generated a total of 45,101,102 (5,682,738,852 bases) and 34,715,846 (4,374,196,596 bases) raw reads, respectively (Table 1). After the cleansing step with an NGS QC Toolkit and removal of low quality (Q < 20) reads, 39,932,160 (5,006,434,933 bases) and 31,353,144 (3,932,946,324 bases) remained as clean reads, respectively (Table 1). The percentages of clean reads were 88.1% and 89.9%, respectively (Table 1). All the clean reads were submitted to the Trinity for de novo assembly. Unigenes were identified after removing redundant sequences from assembled transcripts. The number of unigenes was 104,348, the total length and the average length of the unigenes were 88,123,224 bp and 845 bp, respectively (Table 1). The length distribution of unigenes is presented in Figure 1. Among these unigenes, 66,204 unigenes (63.4%) were no more than 500 bp. A total of 15,382 unigenes (14.7%) were 501–1000 bp, 6991 unigenes (6.7%) were 1001–1500 bp, 4727 unigenes (4.5%) were 1501–2000 bp, 3332 unigenes (3.2%) were 2001–2500 bp, and 7712 unigenes (7.4%) were longer than 2500 bp.

### 3.2. The Most Abundantly Expressed Gene in the Transcriptome Profile

To estimate gene expression levels, we calculated the abundance of reads in the transcriptome. The top 20 most highly expressed transcripts are shown in Table 2. Commonly, the most abundant genes in both the mock and NNV-infected groups were ribosomal proteins, such as ribosomal protein (RPS) 15a, RPL39, RPS28, RPS14, RPLS2, RPS27a, RPL21 and RPL32 essential for biological metabolism. Ubiquitin-like protein 4a (UBL4a), C-C motif chemokine 2 (CCL2), lysozyme g (LYG_EPICO) and two novel genes (ID: SGU016297, SGU008676) were highly expressed in the NNV-infected group compared to that in the mock group. Of them, Ubiquitin-like protein 4a was the most abundant gene in the NNV-infected group.

### 3.3. Functional Annotations

Putative annotations of these transcripts were performed using BlastX as mentioned in the method section. After gene annotation by using BlastX against the non-redundant (nr) database, the putative functions of 43,280 sequences (41.5%) of 104,348 unigenes sequences were identified.

### 3.4. Immune Relevant DEGs Involved in NNV Infection

A total of 3418 unigenes were differentially expressed based on DEG analysis using the TCC package. A total of 372 genes from the total of 3418 DEGs were annotated (Table S1). Immune relevant DEGs were further manipulated manually (Table 3). In immune relevant genes, a variety of cytokines were intensely up-regulated after NNV infection.

Several cytokine genes induced by NNV infection belonged to the chemokine family, including *C-C motif Chemokine Ligand 2* (*CCL2*), *CCL34*, *CCL19*, *CCL4*, *C-X-C motif chemokine ligand 13* (*CXCL13*), *CXCL6*, *CXCL8*, *CXCL9*, *Interleukin-12 subunit alpha* (*IL12A*) and *beta* (*IL12B*), and *IL18B*. *CCL2* was the most critically expressed gene in the infected group showing 10.66 Log Fold Change (FC). *CCL2* is involved in neuroinflammatory processes taking place in the central nervous system in various diseases [28].

Cathepsins are lysosomal cysteine enzymes with important roles in cellular homeostasis and innate immune response [29]. Among a dozen members of the Cathepsin family, subtypes L, H, K, O, S and Z were up-regulated in the brain of sevenband grouper after NNV infection. Specifically, Cathepsin L was highly expressed in the NNV-infected group showing 8.3 Log FC. 

Several lectins were expressed in higher levels in the NNV-infected group compared to the mock group, including C-type lectins (CLEC4M, CLEC10A), galectins (LGALS9, LGALS3), fucolectin (FUCL4), and mannose-binding lectin (MBL). In the case of C-type lectins, its receptor (CD209) was also highly expressed in the infected group (Table 3) indicating that C-type lectin might play specific roles in the response of sevenband grouper to NNV infection.

As expected, a number of antiviral proteins also showed high levels of expression in the NNV-infected group. For example, radical S-adenosyl methionin domain-containing protein 2 (RSAD2), also known as viperin, was highly expressed in the NNV-infected group with 10.40 Log FC. *Mx* gene (*MX*), one of the important downstream effectors of interferon (IFN), was also expressed more in the infected group with 8.64 Log FC. Besides Mx, a lot of IFN-induced proteins were upregulated by NNV infection, including IFN-induced protein 44 (IFI44), IFN-induced protein with tetratricopeptide repeats 5 (IFIT5), IFN-induced very large GTPase 1 (GVINP1), IFN-induced double-stranded RNA-activated protein kinase (EIF2Ak2), and IFN-induced helicase C domain-containing protein 1 (IFIH1). Interestingly, of the various heat shock proteins (HSPs), only HSP30 was significantly upregulated in the NNV-infected group with Log 8.42 FC. NK-Lysin, a known antibacterial protein, was also highly expressed in the NNV-infected group with 8.85 Log FC.

### 3.5. GO Enrichment of Differentially Expressed Genes

**Figure 2:**
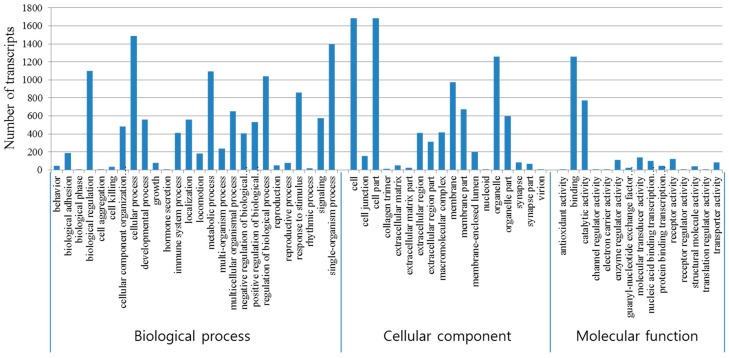
GO annotation of Differentially Expressed Genes (DEGs).

GO is a widely used method to classify gene functions and their products in organisms. Therefore, the identified DEGs were subsequently used for GO enrichment analysis. According to GO terms, 2094 (61.3%) of the total of 3418 DEGs were classified into the three categories of molecular function, biological process, and cellular component. “Binding” (1258 genes, 46.3%) was the major subcategory in the molecular function. The largest subcategory found in the biological process category was “cellular process” (1488 genes, 12.3%) while “Cell” (1687 genes, 19.6%) and “cell part” (1687 genes, 19.6%) were the most abundant GO terms in the cellular component category (Figure 2). Because one gene could be categorized into several subcategories, the sum of genes in the subcategories could exceed 100%. GO analysis of the transcriptome revealed nine molecular function subcategories, 62 biological process subcategories, and 12 cellular component subcategories with *p* value of less than 1 × 10^−5^) (Table S2).

## 4. Discussion

NNV infection has caused high mortalities of sevenband groupers in aqua-farms during the summer season, especially at larval and juvenile stages. It has also caused tremendous economic losses [1]. Due to the greater damage to the sevenband grouper industry, investigation on the molecular response of NNV infection is required to understand the outbreak mechanism of disease and develop prevention methods such as vaccines. In this study, we performed a transcriptome analysis of the brain tissue of sevenband grouper infected with NNV compared to mock brain tissue using a RNA-Seq.

The total number of unigenes and the average length of the unigenes were 104,348 and 845 bp, respectively. The number and average length found in this study indicated a fairly good performance compared to other previous NGS transcriptome studies on crimson spotted rainbowfish (107,749 transcripts, 961 bp) [30] common carp (130,292 transcripts, 1401 bp) [18], blunt snout bream (253,439 transcripts, 998 bp) [31], orange-spotted grouper (116,678 transcripts, 685 bp) [32] and Asian seabass (89,026 transcripts, 1175 bp) [33].

Gene annotation by BlastX provides valuable information about the transcripts. In this study, 43,289 unigenes (41.5%) of 104,348 unigenes were annotated. This is similar to the result of orange-spooted grouper (45.8%) [32]. Liu et al. have addressed the possible reasons of such poor annotation: (1) novel genes; (2) sequencing errors; and (3) artefacts by assembly algorithm [33]. Therefore, more genetic studies are needed to understand the biological functions.

The importance of innate defense mechanisms against viral infection has been extensively reviewed [34,35,36]. In this study, we identified innate immune response relevant genes of sevenband grouper involved in NNV infection. Chemokines are critical components of the immune system. The roles of chemokines and their receptors in viral interactions have been reported in various studies [37]. The chemokines family comprises four subfamilies based on N-terminal cystein-motifs: C, C-C, C-X-C, and C-X3-C subfamilies [38]. In this study, we also detected significant up-regulation of *CCL2*, *CCL34*, *CCL19*, *CCL4*, *CXCL13*, *CXCL6*, *CXCL8*, and *CXCL9* in sevenband grouper brain tissue after NNV infection. Especially, *CCL2* was highly over expressed at about 10.66 Log FC. CCL2 is a pro-inflammatory chemokine that is induced during several human acute and chronic viral infections, including human immunodeficiency virus (HIV) [39], hepatitis C virus [40], Epstein–Barr virus [41], respiratory synctitial virus [42], Severe Acute Respiratory Syndrome (SARS) [43], herpes simplex virus-1 [44], and Japanese encephalitis virus [45].

Cathepsins are lysosomal cysteines that play important roles in normal metabolism for the maintenance of cellular homeostasis. Cathepsins are one of the superfamilies involved in the regulation of antigen presentation and degradation as well as immune responses, including apoptosis, inflammation, and regulation of hormone processing [46,47,48]. In addition, Chandran et al. have shown that Cathepsin B and Cathepsin L are involved in Ebola virus infection [49]. They are involved in the entry of reovirus [50]. Recently, Cathepsin L has also been shown to be involved in the entry mediated by the SARS coronavirus spike glycoprotein [51] as well as in the process of fusion glycoprotein of Hendra virus [52]. In this study, Cathepsin L and Cathepsin S were found to be notably expressed after NNV infection. Their functional roles in the interaction between grouper and NNV merit further studies.

Lectins are carbohydrate-binding proteins that are highly specific for sugar moieties. They mediate the attachment and binding of viruses to their targets [53]. Lectins are also known to play important roles in the immune system. Within the innate immune system, lectins can help mediate the first-line of defense against invading microorganisms. In this study, several kinds of lectins were found to be highly induced in sevenband grouper brain tissue by NNV infection, such as C-type lectins (CTLs), galectins, fucolectin, and mannose-binding lectin. CTLs are the most well studied lectins. They can promote antibacterial and antiviral immune defense [54]. Many CTLs have been identified in teleost but the exact function of CTLs remains unclear.

Hundreds of interferon stimulated genes (ISGs) were transcribed in sevenband grouper brain tissue during NNV infection. Interferon induced protein 44 (IFI44) was expressed the most. IFI44 is an interferon-alpha inducible protein associated with infection of several viruses. Power et al. have demonstrated that IFI44 can inhibit HIV-1 replication in vitro by suppressing HIV-1 LTR promoter activity [55]. Carlton-Smith and Elliott have screened ISGs related to *Bunyamwera orthbunyavirus* replication using nonstructural (NSs) protein knock out virus. One of these ISGs that have inhibitory activity is found to be *IFI44* [56]. Whether protein B2 of NNV has roles in virus replication and its relationship with ISGs such as IFI44 merit further study.

HSPs are one of the most phylogenetically conserved classes of proteins with critical roles in maintaining cellular homeostasis and protecting cells from various stresses [57]. Ironically, HSP70 has roles to suppress some virus infections, and support their replication in other viruses [58]. In this study, *HSP30* was the highly induced gene instead of *HSP70*. *HSP30* has also been reported to be the most induced gene in the NNV infected Asian seabass epithelial cell [33]. However, the function of *HSP30* in NNV infection remains unclear.

Krasnov et al. previously reported the effects of NNV on gene expression in Atlantic cod brain using a microarray [59]. Compared to our study, a number of genes show a similar up-regulation result in the study, such as Caspase, Cathepsins, IRF, Radical *S*-adenosyl methionine domain-containing protein, tripartite motif-containing protein (TRIM) and so on. However, a lot of novel genes (e.g., NK-Lysin, Ubiquitin-like protein 4, Granzyme A, etc.) were identified from our RNA-Seq result because a microarray can only evaluate the genes on a chip.

Our findings are preliminary based on the small scale of the study and the results have not yet been confirmed by an independent technique such as quantitative polymerase chain reaction (qPCR). In future studies, it will be necessary to confirm the expression level of genes and to characterize the function of genes that are highly involved in NNV infection.

## 5. Conclusions 

In conclusion, to the best of our knowledge, this is the first study reporting the transcriptome of brain tissue of NNV-infected sevenband grouper. In this study, we obtained the transcriptome of sevenband grouper. A total of 104,348 transcripts were obtained, including 628 DEGs between NNV infected and non-infected sevenband grouper. A large number of differential expressed genes relevant to immune response were identified as well as several candidate genes (*CCL2, Cathepsins, Lectins, Hsp30,* and *Interferon-induced protein 44*) that were intensely induced by NNV. Their functions in sevenband grouper against NNV infection merit further study. The acquired data from such transcriptome analysis provide valuable information for future functional genes related to NNV infection and VNN outbreak.

## Figures and Tables

**Table 1:** Sequencing, assembly and annotation of transcriptome.

**A. Sequencing and Preprocessing**		
**Sample Type**	**Not Infected (Mock)**	**NNV Infected**
Number of raw reads	45,101,102	34,715,846
Total number of raw bases	5,682,738,852	4,374,196,596
Number of clean reads	39,932,160 (88.5%)	31,353,144 (90.3%)
Total number of clean bases	5,006,434,933 (88.1%)	3,932,946,324 (89.9%)
**B. De novo Assembly and Annotation**		
Number of unigenes		104,348
Total bases		88,123,224
Average length of unigenes		845 bases
Annotation by BLAST		43,280 (41.5%)

**Table 2:** The top 20 most abundant unigenes.

No	Mock			Infected		
	ID	Name	Description	ID	Name	Description
1	SGU067144	-	Hypothetical protein	SGU020577	UBL4a	Ubiquitin-like protein 4a
2	SGU067145	-	Hypothetical protein	SGU005369	-	Hyphothetical protein
3	SGU004764	*EPD1*	Ependymin-1	SGU067144	-	Hyphothetical protein
4	SGU023233	*MT-CO1*	Cytochrome c oxidase subunit 1	SGU067145	-	Hyphothetical protein
5	SGU005369	-	Hypothetical protein	SGU051992	RPS15a	40S ribosomal protein S15a
6	SGU023234	*MT-ND5*	NADH dehydrogenase subunit 5	SGU028675	RPL38	60S ribosomal protein L38
7	SGU051992	*RPS15a*	40S ribosomal protein S15a	SGU004764	EPD1	Ependymin-1
8	SGU028388	*CD59*	CD59 glycoprotein	SGU035083	RPL39	60S ribosomal protein L39
9	SGU025576	*HBA*	Hemoglobin subunit alpha	SGU023233	MT-CO1	Cytochrome c oxidase subunit 1
10	SGU035083	*RPL39*	60S ribosomal protein L39	SGU036307	CCL2	C-C motif chemokine 2
11	SGU021087	*UBIQP_XENLA*	Polyubiquitin	SGU053409	LYG_EPICO	Lysozyme g
12	SGU002473	*RPS28*	40S ribosomal protein S28	SGU002473	RPS28	40S ribosomal protein S28
13	SGU002418	*FABP7*	Fatty acid-binding protein, brain	SGU021087	UBIQP_XENLA	Polyubiquitin
14	SGU028675	*RPL38*	60S ribosomal protein L38	SGU037323	RPL29	60S ribosomal protein L29
15	SGU040127	*RPS14*	40S ribosomal protein S14	SGU003425	RPS27a	40S ribosomal protein S27a
16	SGU028033	*RPLP2*	60S acidic ribosomal protein P2	SGU016297	-	-
17	SGU026642	*HBB2*	Hemoglobin subunit beta-2	SGU005492	UBA52	Ubiquitin-60S ribosomal protein L40
18	SGU003425	*RPS27a*	40S ribosomal protein S27a	SGU034075	RPL21	60S ribosomal protein L21
19	SGU034075	*RPL21*	60S ribosomal protein L21	SGU040127	RPS14	40S ribosomal protein S14
20	SGU003965	*RPL32*	60S ribosomal protein L32	SGU008676	-	-

**Table 3:** Immune relevant differentially expressed genes (DEGs) after NNV infection.

Name	Description	Expression Level (FPKM)		Log FC	*p*-Value	FDR
		Mock	Infected			
**Cytokine**						
*CCL2*	C-C Motif Chemokine 2	51.7	83,844.2	10.66	2.3 × 10^−6^.	6.0 × 10^−3^
*IL12B*	Interleukin-12 Subunit Beta	12.5	14,158.0	10.15	5.6 × 10^−6^	7.1 × 10^−3^
*CCL34A.4*	Chemokine (C-C Motif) Ligand 34a, Duplicate 4	5.8	7038.8	10.24	6.2 × 10^−6^	7.4 × 10^−3^
*CXCL13*	C-X-C Motif Chemokine Ligand 13	10.0	7500.3	9.55	1.4 × 10^−5^	1.3 × 10^−2^
*CCL19*	C-C Motif Chemokine 19	15.0	4523.8	8.24	8.2 × 10^−5^	3.0 × 10^−2^
*CXCL6*	C-X-C Motif Chemokine Ligand 6	60.8	11,405.2	7.55	1.8 × 10^−4^	5.3 × 10^−2^
*IL18R1*	Interleukin-18 Receptor 1	9.2	1814.2	7.63	2.3 × 10^−4^	6.0 × 10^−2^
*CXCL8*	C-X-C Motif Chemokine Ligand 8 (Interleukin-8)	6.7	1307.0	7.61	2.7 × 10^−4^	6.4 × 10^−2^
*EBI3*	Interleukin-27 Subunit Beta	9.2	1506.8	7.36	3.4 × 10^−4^	7.5 × 10^−2^
*CXCL9*	C-X-C Motif Chemokine Ligand 9	12.5	1438.8	6.85	6.4 × 10^−4^	1.2 × 10^−1^
*CCL4*	C-C Motif Chemokine 4	11.7	1328.8	6.83	6.7 × 10^−4^	1.2 × 10^−1^
*IL1R2*	Interleukin-1 Receptor Type 2	6.7	458.6	6.10	2.7 × 10^−3^	2.9× 10^−1^
*CXCR4*	C-X-C Chemokine Receptor Type 4	35.0	1016.3	4.86	8.4 × 10^−3^	6.1 × 10^−1^
*CRLF1*	Cytokine Receptor-like Factor 1	9.2	326.3	5.15	9.1 × 10^−3^	6.5× 10^−1^
*XCR1*	Chemokine Xc Receptor 1	0.8	97.5	6.88	1.0 × 10^−2^	7.0 × 10^−1^
*IL13RA1A*	Il-13 Receptor-alpha-1-a Precursor	117.5	2426.3	4.37	1.4 × 10^−2^	8.6 × 10^−1^
*CXCR3*	C-X-C Chemokine Receptor Type 3	56.7	1140.0	4.33	1.6 × 10^−2^	9.3 × 10^−1^
*IL12A*	Interleukin-12 Subunit Alpha	3.5	128.3	5.20	1.8 × 10^−2^	9.9 × 10^−1^
**Cathepsin**						
*CTSL*	Cathepsin L	7.5	2370.0	8.30	9.2 × 10^−5^	3.2 × 10^−2^
*CTSH*	Cathepsin H	418.3	9396.3	4.49	1.1 × 10^−2^	7.5 × 10^−1^
*CTSK*	Cathepsin K	170.0	8802.5	5.69	2.3 × 10^−3^	2.7 × 10^−1^
*CTSO*	Cathepsin O	20.0	485.0	4.60	1.4 × 10^−2^	8.6 × 10^−1^
*CTSS*	Cathepsin S	388.3	20,089.4	5.69	2.2 × 10^−3^	2.7 × 10^−1^
*CTSZ*	Cathepsin Z	604.2	10,606.8	4.13	1.8 × 10^−2^	9.8 × 10^−1^
**Cluster of differentiation**						
*CD274*	Programmed Cell Death 1 Ligand 1	5.8	2255.0	8.60	6.7 × 10^−5^	2.8 × 10^−2^
*CD4*	T-cell Surface Glycoprotein CD4	3.3	256.9	6.27	3.7 × 10^−3^	3.6 × 10^−1^
*CD209A*	CD209 Antigen-like Protein A	3.3	251.6	6.24	3.9 × 10^−3^	3.8 × 10^−1^
*CD48*	CD48 Antigen	30.8	1969.6	6.00	1.7 × 10^−3^	2.3 × 10^−1^
*CD209D*	CD209 Antigen-like Protein D	68.3	3346.3	5.61	2.7 × 10^−3^	3.0 × 10^−1^
*TSPAN6*	Tetraspanin-6	9.7	387.8	5.32	7.2 × 10^−3^	5.6 × 10^−1^
**Complement**						
*C4A*	Complement C4-a	9.6	2062.4	7.74	2.0 × 10^−4^	5.6 × 10^−2^
*C1QA*	Complement C1q Subcomponent Subunit A	74.2	5517.8	6.22	1.1 × 10^−3^	1.8 × 10^−1^
*C4*	Complement C4	1.2	220.1	7.53	1.3 × 10^−3^	1.9 × 10^−1^
*C1QC*	Complement C1q Subcomponent Subunit C	61.7	4210.0	6.09	1.4 × 10^−3^	2.0 × 10^−1^
*C1S*	Complement C1s Subcomponent	126.4	8272.5	6.03	1.4 × 10^−3^	2.0 × 10^−1^
*C1QB*	Complement C1q Subcomponent Subunit B	116.7	7135.2	5.93	1.7 × 10^−3^	2.2 × 10^−1^
*C7*	Complement Component C7	30.0	1288.2	5.42	3.9 × 10^−3^	3.8 × 10^−1^
*CFB*	Complement Factor B	144.2	4880.0	5.08	5.3 × 10^−3^	4.6 × 10^−1^
**Lectin**						
*CLEC4M*	C-type Lectin Domain Family 4 Member M	10.0	5250.0	9.04	2.9 × 10^−5^	1.9 × 10^−2^
*LGALS9*	Galectin-9	89.2	8098.8	6.50	7.6 × 10^−4^	1.3 × 10^−1^
*FUCL4_ANGJA*	Fucolectin-4	32.5	1838.8	5.82	2.2 × 10^−3^	2.7 × 10^−1^
*CLEC10A*	C-type Lectin Domain Family 10 Member A	12.5	603.8	5.59	4.1 × 10^−3^	3.9 × 10^−1^
*MBL*	Mannose-binding Lectin	1.7	175.0	6.71	4.2× 10^−3^	4.0 × 10^−1^
*LGALS3*	Galectin-3	10.8	415.0	5.26	7.1× 10^−3^	5.5 × 10^−1^
*LGALS3BPA*	Galectin-3-binding Protein A	1083.3	27,214.6	4.65	8.9 × 10^−3^	6.5 × 10^−1^
**Ubiquitination**						
*UBL4A*	Ubiquitin-like Protein 4a	31.7	87,332.8	11.43	8.0 × 10^−7^	6.0 × 10^−3^
*HERC5*	E3 Ubiquitin-protein Ligase Herc5	3.1	4851.1	10.61	5.6 × 10^−6^	7.1 × 10^−3^
*HERC6*	E3 Ubiquitin-protein Ligase Herc6	65.0	34,045.8	9.03	2.2 × 10^−5^	1.6 × 10^−2^
*USP18*	Ubiquitin Carboxyl-terminal Hydrolase 18	14.2	4250.9	8.23	8.4 × 10^−5^	3.0 × 10^−2^
*USP12*	Ubiquitin Carboxyl-terminal Hydrolase 12	9.2	1986.2	7.76	1.9 × 10^−4^	5.4 × 10^−2^
*UBR1*	E3 Ubiquitin-protein Ligase Ubr1	18.4	2933.8	7.32	2.9 × 10^−4^	6.6 × 10^−2^
*TRIM21*	Tripartite Motif-containing Protein 21	6.7	1061.3	7.31	4.2 × 10^−4^	8.7 × 10^−2^
*TRIM47*	Tripartite Motif-containing Protein 47	9.2	1332.5	7.18	4.4 × 10^−4^	8.8 × 10^−2^
*RNF213*	E3 Ubiquitin-protein Ligase Rnf213	40.8	4928.8	6.92	4.6 × 10^−4^	8.9 × 10^−2^
*TRIM29*	Tripartite Motif-containing Protein 29	58.0	2261.1	5.29	4.3 × 10^−3^	4.0 × 10^−1^
*TRIM39*	Tripartite Motif-containing Protein 39	7.0	354.2	5.67	4.7 × 10^−3^	4.2 × 10^−1^
*TRIM25*	Tripartite Motif-containing Protein 25	2.5	182.5	6.19	5.8 × 10^−3^	4.8 × 10^−1^
*HERC4*	E3 Ubiquitin-protein Ligase Herc4	788.6	17,971.6	4.51	1.1 × 10^−2^	7.3 × 10^−1^
*TRIM16*	Tripartite Motif-containing Protein 16	17.5	476.7	4.77	1.2 × 10^−2^	7.7 × 10^−1^
*TRIM14*	Tripartite Motif-containing Protein 14	85.7	1728.9	4.33	1.5 × 10^−2^	8.9 × 10^−1^
**Others**						
*RSAD2*	Radical S-adenosyl Methionine Domain-containing Protein 2	30.8	41,672.6	10.40	3.4 × 10^−6^	7.1 × 10^−3^
*IFI44*	Interferon-induced Protein 44	62.8	73,980.2	10.20	4.3 × 10^−6^	7.1 × 10^−3^
*IFIT5*	Interferon-induced Protein With Tetratricopeptide Repeats 5	4.2	3119.8	9.55	2.0 × 10^−5^	1.6 × 10^−2^
*SOCS1*	Suppressor Of Cytokine Signaling 1	25.8	13,362.6	9.01	2.5 × 10^−5^	1.7 × 10^−2^
*MX*	Interferon-induced GTP-binding Protein Mx	49.2	19,664.4	8.64	3.9 × 10^−5^	2.3 × 10^−2^
*NKL*	Antimicrobial Peptide Nk-lysin	8.3	3838.8	8.85	4.0 × 10^−5^	2.3 × 10^−2^
*FCGR1A*	High Affinity Immunoglobulin Gamma Fc Receptor I	33.3	12,632.2	8.57	4.5 × 10^−5^	2.5 × 10^−2^
*FCER1A*	High Affinity Immunoglobulin Epsilon Receptor Subunit Alpha	13.4	5255.2	8.62	4.8 × 10^−5^	2.5 × 10^−2^
*PIGR*	Polymeric Immunoglobulin Receptor	5.0	2332.5	8.87	4.9 × 10^−5^	2.5 × 10^−2^
*LYG_EPICO*	Lysozyme G	352.5	120,945.0	8.42	5.0 × 10^−5^	2.5 × 10^−2^
*SAMD9*	Sterile Alpha Motif Domain-containing Protein 9	2.5	1271.2	8.99	6.5 × 10^−5^	2.8 × 10^−2^
*IRF4*	Interferon Regulatory Factor 4	10.0	2811.3	8.14	1.1 × 10^−4^	3.6 × 10^−2^
*GVINP1*	Interferon-induced Very Large GTPase 1	53.3	11,420.6	7.74	1.4 × 10^−4^	4.5 × 10^−2^
*TMEM173*	Stimulator of Interferon Genes Protein	23.3	4917.5	7.72	1.6 × 10^−4^	4.8 × 10^−2^
*MPEG1*	Macrophage-expressed Gene 1 Protein	13.3	2433.8	7.51	2.4 × 10^−4^	6.0 × 10^−2^
*HSP30*	Heat Shock Protein 30	1.7	572.5	8.42	2.4 × 10^−4^	6.0 × 10^−2^
*GZMA*	Granzyme A	9.2	1761.4	7.57	2.4 × 10^−4^	6.0 × 10^−2^
*IRF3*	Interferon Regulatory Factor 3	5.8	1155.6	7.63	2.8 × 10^−4^	6.6 × 10^−2^
*EIF2AK2*	Interferon-induced, Double-stranded RNA-activated Protein Kinase	15.6	2438.5	7.29	3.3 × 10^−4^	7.3 × 10^−2^
*IRF1*	Interferon Regulatory Factor 1	349.2	22,350.8	6.00	1.5 × 10^−3^	2.0 × 10^−1^
*PSMB6L-B*	Proteasome Subunit Beta Type-6-b Like Protein	131.7	8506.1	6.01	1.5× 10^−3^	2.0 × 10^−1^
*CASP3*	Caspase-3	24.2	1688.8	6.13	1.5 × 10^−3^	2.1 × 10^−1^
*PSME1*	Proteasome Activator Complex Subunit 1	220.8	12,675.8	5.84	1.8 × 10^−3^	2.4 × 10^−1^
*IFIH1*	Interferon-induced Helicase C Domain-containing Protein 1	96.7	5280.5	5.77	2.1 × 10^−3^	2.6 × 10^−1^
*PSMB8*	Proteasome Subunit Beta Type-8	86.7	3997.5	5.53	3.0 × 10^−3^	3.2 × 10^−1^
*GRN*	Granulins	275.0	11,720.0	5.41	3.3 × 10^−3^	3.3 × 10^−1^
*MR1*	Major Histocompatibility Complex Class I-related Gene Protein	153.6	6162.8	5.33	3.8 × 10^−3^	3.6 × 10^−1^
*SOCS3*	Suppressor Of Cytokine Signaling 3	125.7	4607.5	5.20	4.6 × 10^−3^	4.2 × 10^−1^
*IGSF3*	Immunoglobulin Superfamily Member 3	11.7	408.0	5.13	8.3 × 10^−3^	6.1 × 10^−1^
*IRGC*	Interferon-inducible GTPase 5	11.9	346.9	4.86	1.2 × 10^−2^	7.7 × 10^−1^

Note, FPKM: fragments per kilobase of transcript per million mapped reads; Log FC: Log value of fold changes, FDR: false discovery rate.

## Supplementary Materials

The following are available online at www.mdpi.com/2073-4425/8/1/31/s1, Table S1: The annotated DEGs, Table S2: Gene ontology of sevenband grouper transcriptome after NNV infection.
